# Bloodstream infections caused by ST2 *Acinetobacter baumannii*: risk factors, antibiotic regimens, and virulence over 6 years period in China

**DOI:** 10.1186/s13756-020-00876-6

**Published:** 2021-01-18

**Authors:** Kaihang Yu, Weiliang Zeng, Ye Xu, Wenli Liao, Wenya Xu, Tieli Zhou, Jianming Cao, Lijiang Chen

**Affiliations:** 1 Department of Clinical Laboratory, the First Affiliated Hospital of Wenzhou Medical University, Wenzhou, 325000 China; 2 School of Laboratory Medicine and Life Science, Wenzhou Medical University, Wenzhou, 325000 China

**Keywords:** *Acinetobacter baumannii*, Bloodstream infection, Multidrug-resistant, Cefoperazone/sulbactam, Tigecycline, ST2

## Abstract

**Background:**

Bloodstream infection (BSI) caused by multidrug-resistant *Acinetobacter baumannii* (MDR-AB) has been increasingly observed among hospitalized patients. The following study analyzed the epidemiology and microbiological characteristics of MDR-AB, as well as the clinical features, antimicrobial treatments, and outcomes in patients over a six years period in China.

**Methods:**

This retrospective study was conducted in a large tertiary hospital in China between January 2013 and December 2018. The clinical and microbiological data of all consecutive hospitalized patients with MDR-AB induced bloodstream infection were included and analyzed.

**Results:**

A total of 108 BSI episodes were analyzed. All MDR isolates belonged to ST2, a sequence type that has spread all over the world. Overall, ST2 strains showed strong biofilm formation ability, high serum resistance, and high pathogenicity. As for the clinical characteristics of the patient, 30-day mortality was 69.4% (75/108). The three main risk factors included mechanical ventilation, intensive care unit (ICU) stay, and thrombocytopenia; three protective factors included a change of antimicrobial regimen within 48 h after positive blood culture, use of the antibacterial agent combination, and more inpatient days. The most effective antibacterial regimen was the combination of cefoperazone/sulbactam and tigecycline.

**Conclusions:**

BSI caused by ST2 *A.baumannii* represents a difficult challenge for physicians, considering the high mortality associated with this infection. The combination of cefoperazone/sulbactam and tigecycline may be an effective treatment option.

## Background

*A. baumannii* has become the leading cause of bloodstream infections (BSI) among hospitalized patients [1]. These bacteria are usually resistant to antibiotics; thus, treatment options are very limited [2].

Carbapenem antibiotics are the first-line drugs used to treat *A. baumannii* infections [3]. However, *A. baumannii* resistance to carbapenem antibiotics has rapidly increased over recent years [4]. According to China Antimicrobial Resistance Surveillance Network (CHINET) program, the resistance rate of *A. baumannii* to imipenem and meropenem increased to 62.8% and 59.4% in 2013 and to 77.1% and 78.1% in 2018, respectively (http://www.chinets.com/). Carbapenem-resistant *A. baumannii* (CRAB) are typically multidrug-resistant strains that are usually sensitive to tigecycline and polymyxins [5].

According to World Health Organization (WHO), MDR-AB has recently been considered the most critical pathogen for public health, topping the global priority list of antibiotic-resistant bacteria [6]. MDR-AB population is characterized by clonal dissemination, as revealed by multi-locus sequence typing (MLST) [7], and one of the most successful clonal lineages, sequence type 2, is associated with nosocomial outbreaks and multidrug-resistance and has spread globally [8]. The clinical performance of tigecycline and polymyxins falls short due to their unfavourable pharmacokinetics and toxicity. Therefore, there is still no optimal therapeutic regimen for MDR-AB infection [9]. A study conducted in Italy showed that carbapenem or polymyxins-based regimens were the main options for treating bloodstream infections caused by MDR-AB [10]. In China, sulbactam-based regimens, tigecycline-based regimens, and polymyxins-based regimens have been recommended as the main treatment for MDR-AB [11]. However, these recommendations are based on small-scale retrospective studies, lacking systematic and comprehensive clinical research evidence. Large-scale clinical randomized controlled trials have not been evaluated in patients with MDR-AB infection. In addition, polymyxins, which is commonly used for the treatment of Gram-negative bacterial infection, has not been standardized in China until 2019, resulting in a low usage in clinicians [12]. Therefore, sulbactam and tigecycline are currently the main clinical treatments for MDR-AB, but the efficacy of the two regimens remains to be clarified.

In addition to its multidrug resistance, *A. baumannii* also has various virulence factors. These factors, including outer membrane protein A [13], lipopolysaccharide [14], capsular polysaccharide [15], phospholipase D [16], outer membrane vesicle [17] and acinetobactin-mediated iron acquisition system [18] help *A. baumannii* evade the host immune response and improve survival in the human serum. The ability of biofilm formation not only contributes to easy survival and transfer of *A. baumannii* in the hospital environment, such as attached to various biotic and abiotic surfaces [19], but also enhances the pathogenicity in mammalian septicemia model [20].

High resistance and virulence of bacteria are the main factors associated with high mortality in patients with bloodstream infections [10, 21]. Some studies reported that the patient’s condition, previous exposure to antimicrobial (especially broad-spectrum antibiotics), previous colonization with *A. baumannii*, increased Pitt bacteremia score, being in the intensive care unit (ICU), and recent invasive procedures are risk factors associated with MDR acquisition in *A. baumannii* bloodstream infection [22–24]. Furthermore, compared to sensitive cases, the bloodstream infection caused by MDR-AB usually leads to higher total hospitalization costs [25], significantly impacting the clinical and economic burden.

In this study, we analyzed the clinical features, different antimicrobial treatments, and outcomes in patients with MDR-AB bloodstream infection, as well as the epidemiological and virulence characteristics of these isolates. Our results can help clinicians and infection control specialists in developing successful empirical treatments and infection control measures.

## Methods

### Study location and patient

This study was conducted at the First Affiliated Hospital of Wenzhou Medical University, a 4100-bed teaching hospital (Wenzhou, China), over a 6-year period (1 January 2013 to 31 December 2018). Inclusion criteria of *A. baumannii-*induced bloodstream infection were [10]: (1) age ≥ 18 years; (2) blood culture positive for MDR-AB; (3) clinical signs consistent with infection; (4) the infection occurred ≥ 48 h after hospital admission. Only the first episode was included if the patient had multiple episodes of *A. baumannii-*induced BSI.

### Study design and data collection

A retrospective study design was employed. The main outcome was 30-day in-hospital mortality. Clinical information and laboratory parameters were collected from medical charts that used predefined definitions of variables. The collected data included demographic characteristics, underlying diseases, length of stay in the hospital, treatments and procedures performed, laboratory results, antimicrobial therapies, and all-cause 30-day mortality.

### Strains identification and antimicrobial susceptibility testing

The VITEK-2 automated system (BioMérieux, Marcy-l’Étoile, France) was used for bacteria identification. Minimum inhibitory concentrations (MICs) of 14 antibiotics were tested by the agar dilution method, including imipenem, meropenem, ceftazidime, ceftriaxone, gentamicin, tobramycin, ciprofloxacin, levofloxacin, ampicillin/sulbactam, trimethoprim/sulfamethoxazole, piperacillin/tazobactam, cefperazone/sulbactam, tigecycline and polymyxin B. The results were interpreted according to the recommendations of the Clinical and Laboratory Standards Institute (CLSI; Pittsburgh, PA, USA). The results of tigecycline were interpreted according to the recommendation of the Food and Drug Administration, with MICs of ≤ 2, 4, and ≥ 8 μg/ml interpreted as susceptible, intermediate, and resistant, respectively [26]. MDR was defined as bacteria non-susceptible to three or more different antimicrobial categories; extensively drug-resistant (XDR) was defined as non-susceptibility to at least one agent in all but two or fewer antimicrobial categories. All these strains were stored at − 80 °C for further research.

### Multi-locus sequence typing (MLST)

In the present study, seven housekeeping genes (*cpn60*, *fusA*, *gltA*, *pyrG*, *recA*, *rplB*, and *rpoB*) were amplified and sequenced to characterize the genotypes of all isolates according to the provided protocols (www.pasteur.fr/mlst). The alleles and STs were assigned according to the online database of the Institut Pasteur’s MLST web site of *A. baumannii*.

### Biofilm formation

The biofilm formation ability of the *A. baumannii* isolates on 96-well polystyrene microtiter plates was assessed using the crystal violet staining, as formerly described [27]. The results were classified into the four following categories [28]: non-biofilm producer, weak biofilm producer, medium biofilm producer, and strong biofilm producer.

### Serum resistance assay

Serum sensitivity assays were performed on clinical isolates [29]. The bacterial suspension was mixed with normal human serum (NHS) and incubated at 37 °C. Calculate the colony-forming units (CFUs) of bacteria by single plate-serial dilution spotting (SP-SDS) [30]. All experiments were performed in triplicate, and results were expressed as percent survival.

### Galleria mellonella larva infection assay

The virulence of *A. baumannii* was estimated by infecting *G. mellonella* larvae, as previously described [31]. Briefly, a suspension of *A. baumannii* was prepared in phosphate-buffered saline (PBS). Then, 10 μL of this suspension was injected through the last proleg of each larva. Ten larvae were used for each experiment; experiment was performed 3 times.

### Statistical analysis

All statistical analyses were performed using SPSS 22.0 software (IBM, Armonk, NY, USA). The categorical variables were listed as percentages, and the continuous data were expressed as mean ± standard deviation (mean ± SD) or median (25th-75th percentile). The chi-squared test or Fisher’s exact test was used for categorical variables, and the odds ratio (OR) was calculated with confidence intervals (CIs) of 95%. The continuous data were analyzed using the Student’s *t*-test or Mann–Whitney *U* test. P-value < 0.05 was considered statistically significant. All tests were two-tailed. To determine the risk factors for *A. baumannii-*induced BSI, univariate logistic regression analyses were performed. All variables with a *P-*value < 0.05 were included in the multivariate model.

## Results

### Clinical factors associated with 30-day mortality among patients with ST2 *A. baumannii*-induced BSI

A total of 108 patients with *A. baumannii-*induced BSI were enrolled during the 6-year study period. Seventy-five patients died within 30 days, with a mortality rate of 69.4%. The demographics between the non-survivor group and survivor group were similar, as shown in Table 1. Briefly, the majority of patients in both groups were elderly and male patients. The most common comorbidity was pneumonia. MDR-AB was also isolated from sputum in 86 patients (79.6%) during the hospitalization. Empirical antimicrobial therapy was performed on all patients. The univariate regression analysis revealed that the use of mechanical ventilation (OR 2.49, 95% CI [1.00–6.22]), ICU stay (OR 8.82, 95% CI [3.38–23.02]), and thrombocytopenia (OR 8.72, 95% CI [1.93–39.30]) were all significant risk factors associated with patients mortality (Table 1). Furthermore, deep vein intubation and other microbial isolation were not significantly associated with survival (*P* = 0.247, fungi *P* = 0.890, and bacteria *P* = 0.642, respectively). In contrast, change of antimicrobial agents within 48 h after positive blood culture, the use of the antibacterial agent combination and more inpatient days were significantly associated with survival (*P* < 0.001, *P* = 0.037, and *P* = 0.007, respectively) (Table 1).

**Table 1:** Univariate analysis comparing survivors and non-survivors at 30 days from infection onset

Variables	Nonsurvivor	Survivor	Univariate analysis	
			OR (95% CI)	*P* value
Male sex, (%)	78.7	69.7	1.60 (0.64–4.05)	0.315
Age, median (range)	62 (15–89)	55 (18–89)	–	0.360
Comorbidities, (%)				
Pneumonia	58.7	60.6	0.92 (0.40–2.13)	0.850
Diabetes	17.3	6.1	3.25 (0.69–15.31)	0.208
Liver cirrhosis	4.0	6.1	0.65 (0.10–4.06)	1.000
Malignant tumor	12.0	12.1	0.99 (0.28–3.47)	1.000
Trauma	16.0	24.2	0.60 (0.22–1.63)	0.310
ICU stay, (%)	86.7	42.4	8.82 (3.38–23.02)	**< 0.001**
Inpatient days, median (range)	25 (6–155)	35 (12–163)	–	**0.007**
Deep vein intubation, (%)	89.3	78.8	2.25 (0.74–6.85)	0.247
Mechanical ventilation, (%)	81.3	60.0	2.49 (1.00–6.22)	**0.048**
Polymicrobial infection, (%)				
Fungi	12.0	15.6	0.76 (0.24–2.48)	0.890
Other bacteria	46.7	51.5	0.82 (0.36–1.87)	0.642
*A.baumannii* isolated from other sites during hospital stay, (%)				
Sputum	80.0	78.8	1.08 (0.39–2.95)	0.885
Cerebrospinal fluid	4.0	3.0	1.33 (0.13–13.31)	1.000
Usage of antibiotic, mean (range), (%)				
Number of antibiotic classes used	3.16 (1–6)	3.52 (1–5)	–	0.162
Empirical antimicrobial therapy before isolating from blood	100	100	–	–
Change antimicrobial within 48 h after isolating from blood	53.3	87.9	0.16 (0.05–0.49)	** < 0.001**
Combination therapy	36.0	54.5	0.41 (0.18–0.96)	**0.037**
Laboratory examination, mean ± SD, (%)				
White blood cell count (× 10^9^/L)	13.2 ± 9.70	16.0 ± 11.0	–	0.208
Leukopenia (< 4 × 10^9^/L)	18.7	6.1	3.56 (0.76–16.65)	0.160
Hemoglobin (g/L)	90.5 ± 21.4	94.7 ± 19.3	–	0.328
Anemia (< 100 g/L)	69.3	60.6	1.47 (0.63–3.45)	0.375
Platelet count (× 10^9^/L)	123.1 ± 106.7	201.7 ± 104.0	–	** < 0.001**
Thrombocytopenia (< 50 × 10^9^/L)	36.0	6.1	8.72 (1.93–39.30)	** < 0.001**

Bold values are statistically significant (*P* < 0.05)

Antimicrobial treatment regimens and clinical outcomes of 108 patients are listed in Table 2. During hospitalization, the usage rate of carbapenem, cefoperazone/sulbactam, tigecycline, piperacillin/tazobactam and polymyxin B were 85.2% (92/108), 69.4% (75/108), 45.4% (49/108), 27.8% (30/108) and 7.4% (8/108), respectively. However, the usage of these antimicrobials was not significantly associated with survival (*P* > 0.05). Detailed antimicrobials therapy options after bacteria isolation are listed in Table 2.

**Table 2:** Antibiotic regimens therapy during hospitalization

Antimicrobial	Total (n = 108)	Nonsurvivor (n = 75)	Survivor (n = 33)
Antibiotic usage during hospitalization, n (%)			
Carbapenem	92 (85.2)	66 (88.0)	26 (78.8)
Cefoperazone/sulbactam	75 (69.4)	49 (65.3)	26 (78.8)
Piperacillin/tazobactam	30 (27.8)	18 (24.0)	12 (36.4)
Tigecycline	49 (45.4)	33 (44.0)	16 (48.5)
Polymyxin B	8 (7.4)	4 (5.3)	4 (12.1)
Antibiotic regimens after positive blood culture, n (%)			
Carbapenem	16 (14.8)	12 (16.0)	4 (12.1)
Cefoperazone/sulbactam	14 (13.0)	10 (13.3)	4 (12.1)
Tigecycline	6 (5.6)	6 (8.0)	0 (0)
Carbapenem + Tigecycline	9 (8.3)	9 (12.0)	0 (0)
Cefoperazone/sulbactam + Tigecycline	28 (25.9)	13 (17.3)	15 (45.5)
Polymyxin B + Tigecycline	5 (4.6)	3 (4.0)	2 (6.1)

The most common treatment option was the combination of cefoperazone/sulbactam and tigecycline (25.9%, 28/108), with 53.6% (15/28) survival rate, followed by the monotherapy of carbapenem, cefoperazone/sulbactam and tigecycline (14.8%, 13.0%, and 5.6%, respectively), and the survival rates were 25% (4/16) 28.8% (4/14) and 0% (0/6), respectively. Nine patients were treated by combining carbapenem and tigecycline; however, no patients survived. Among 5 patients treated by combining polymyxin B and tigecycline, 2 patients survived.

### Antimicrobial susceptibility and MLST of *A. baumannii*

The resistance rates of 108 *A. baumannii* were the following: imipenem 100%, meropenem 100%, ceftriaxone 100%, ciprofloxacin 100%, ceftazidime 100%, ampicillin/sulbactam 99.1%, piperacillin/tazobactam 99.1%, gentamicin 98.2%, levofloxacin 94.4%, tobramycin 85.2%, trimethoprim/sulfamethoxazole 76.7%. The resistance rate, intermediate rate, and sensitive rate of cefperazone/sulbactam were 77.8%, 18.5% and 3.7%, and those of tigecycline were 6.5%, 5.5% and 88.0%, respectively (Table 3). Among 61 isolates (56.5%) presented an XDR phenotype, most were sensitive only to polymyxin B and tigecycline. The results of MLST showed that all isolates were ST2.

**Table 3:** The resistance rates of MDR-AB to 14 antimicrobial agents in the years 2013 to 2018 (%)

Antibiotics	Total	2013	2014	2015	2016	2017	2018
Imipenem	100	100	100	100	100	100	100
Meropenem	100	100	100	100	100	100	100
Ceftazidime	100	100	100	100	100	100	100
Ceftriaxone	100	100	100	100	100	100	100
Gentamicin	98.2	100	100	96	100	93.8	100
Tobramycin	85.2	100	93.3	84	92	87.5	53.3
Ciprofloxacin	100	100	100	100	100	100	100
Levofloxacin	94.4	83.3	100	96	92	93.8	100
Ampicillin/sulbactam	99.1	100	100	100	100	93.8	100
Trimethoprim/sulfamethoxazole	76.7	100	13.3	56	52	93.8	100
Piperacillin/tazobactam	99.1	100	100	100	100	93.8	100
Cefperazone/sulbactam	77.8	83.3	66.7	80	80	75	80
Tigecycline	6.5	8.3	6.7	4	8	0	13.3
Polymyxin B	0	0	0	0	0	0	0

### Pathogenicity of *A. baumannii*

**Fig. 1:**
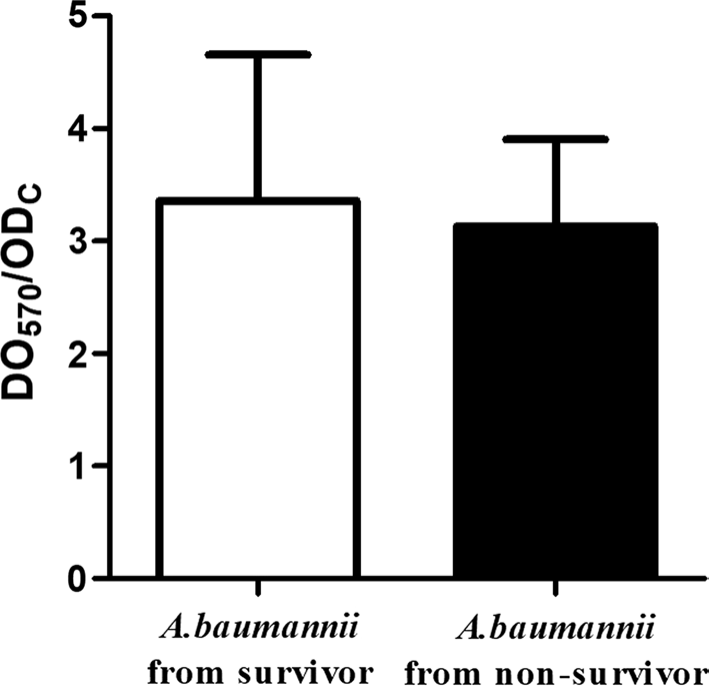
Biofilm formation ability of the 30 *A. baumannii* isolated from the survivors and from the non-survivors

**Fig. 2:**
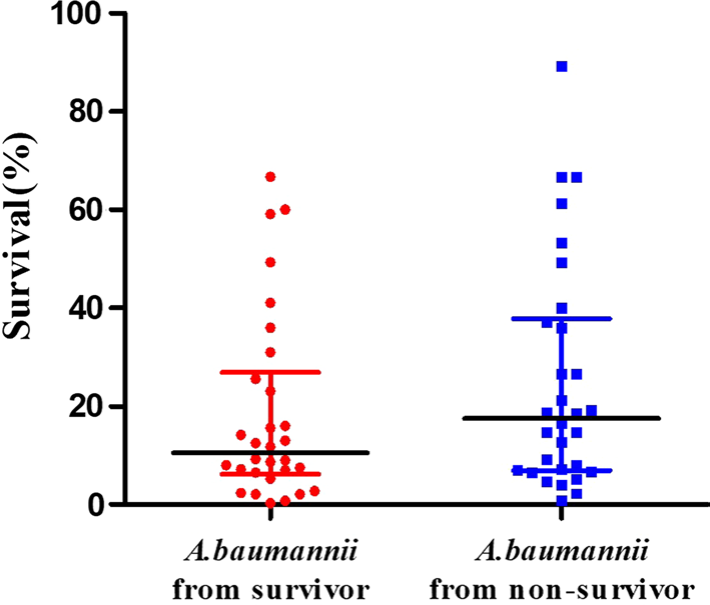
Serum resistance assay of 30 *A. baumannii* isolated from the survivors and 30 from the non-survivors

Thirty *A. baumannii* isolated from the survivors and 30 *A. baumannii* isolated from the non-survivors were randomly selected for biofilm formation experiment and serum resistance assay. Among those who survived, the percent of weak biofilm producer, medium biofilm producer, and strong biofilm producer were 13.3%, 66.7%, and 20%, respectively. Among those who died, the percent of weak biofilm producer, medium biofilm producer, and strong biofilm producer was 6.7%, 83.3%, and 10%, respectively. However, the biofilm formation ability in the two groups was not statistically different (3.36 ± 1.30 vs 3.13 ± 0.77; *P* = 0.417) (Fig. 1). On the other hand, after 3 h incubation with normal human serum, the survival rate of *A. baumannii* isolated from non-survivors was higher than *A. baumannii* isolated from survivors. Still, the survival rates were not statistically different among the two groups (17.5% [6.9%—38.0%] vs. 10.5% [6.2%—27.0%]; *P* = 0.209 (Fig. 2). The survival rate of the standard strain ATCC19606 was less than 0.1%.

**Fig. 3:**
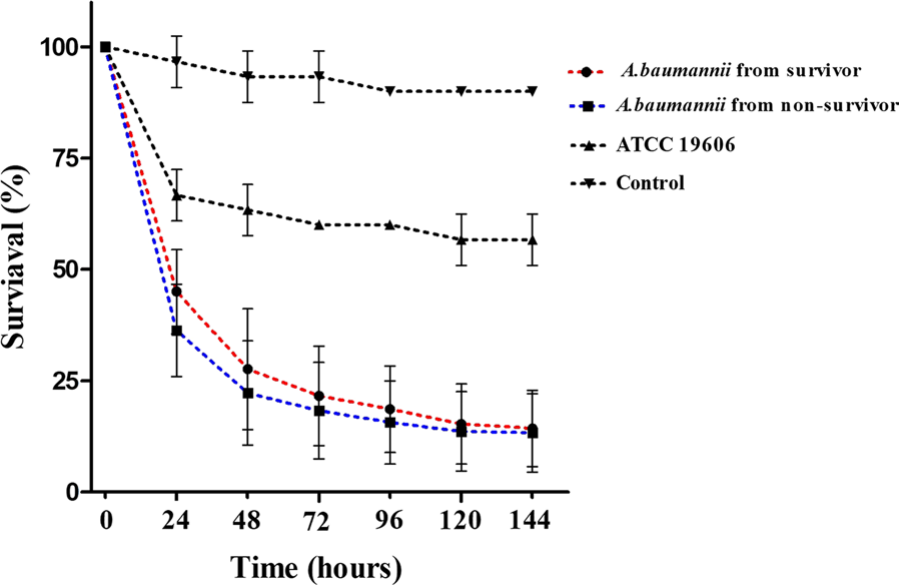
Survival of *G. mellonella* of 10 *A. baumannii* isolated from the survivors and 10 from the non-survivors

Furthermore, 10 *A. baumannii* isolated from the survivors and 10 *A. baumannii* isolated from the non-survivor were randomly selected for *G. mellonella* larva infection assay. *G. mellonella* survival data are shown in Fig. 3. The survival rates of the infected larvae between the two groups were not statistically different in every observation between day 1 and day 6 (*P* = 0.522). The pathogenicity of ST2 was significantly higher than ATCC 19606 (*P* < 0.001).

## Discussion

*A.baumannii* has become a critical hospital-acquired pathogen. Although the bacteria is typically associated with hospital-acquired pneumonia, *A.baumannii* induced bloodstream infection has become a significant health problem [32]. The most alarming fact is the rapid rise in MDR-AB strains, limiting the range of antibiotics available for treatment [33]. Therefore, new strategies are needed to prevent and treat bloodstream infections caused by MDR-AB. It is essential to analyze clonal relationships among pathogens to better understand their molecular epidemiology.

Historically, global clones I and II have been identified as responsible for widespread of MDR-AB across the globe [34]. The analysis of all publicly available genome sequences shows that ST2 (the representative ST type of global clone II), ST1, ST79, and ST25 account for more than 71% of all sequenced genomes [8]. Although it was initially thought that these clones were limited to Europe [35], *A. baumannii* population has been found in more than 30 countries [34], including United States, Italy, Australia, Thailand, United Kingdom, and China. The ST2 *A. baumannii* seems to be widely spread in China. Previous studies have shown that ST2 accounts for 100% of the dominant pulsotypes of CRAB in Heilongjiang Province [36]. Another study from Taiwan Province also showed that 98% of *A. baumannii* belongs to ST2 [37]. Yang et al. found that ST2 occupies a dominant position in 11 different Chinese provinces [38]. However, the MDR-AB population structures isolated from bloodstream infections in this study were all ST2. *A. baumannii,* which are nosocomial pathogens usually transmitted from the medical environment (patient, medical staff, or medical device). Several studies have shown that *A. baumannii* can spread between hospitals [39, 40], which means the prevalence of ST2 is more serious in this region, not just the hospital involved in this study.

The clinical factors associated with 30-day mortality among patients with ST2 *A. baumannii-*induced BSI were also analyzed in this study. The use of mechanical ventilation, ICU stay, and thrombocytopenia have all been associated with higher mortality. Previous studies demonstrated that the mortality risk factors associated with poor prognosis include older age, the severity of the underlying disease, septic shock, a high Pitt bacteremia score, previous surgery, mechanical ventilation, and inappropriate antimicrobial treatment [21, 41, 42]. The ICU has been identified as the unit at the highest risk of the nosocomial outbreak of MDR-AB [43]. Our data also showed that ST2 BSI is a special ICU acquired infection, which may be caused by patient impaired immune function. Thrombocytopenia indicates severe infection, suggesting poor prognosis. Moreover, change of antimicrobial regimen within 48 h after positive blood culture, the use of antibacterial agent combination, and more inpatient days were significantly all associated with survival. A large retrospective study, which was performed across 27 Turkish hospitals [44], analyzed the clinical outcomes of XDR-AB bloodstream infection patients revealing that compared with the single-agent treatment group, the in-hospital mortality rate of the combined treatment group was significantly lower, and the microbial eradication rate was significantly higher than that of the single-agent treatment group. Early appropriate antibacterial therapy is essential to reduce mortality caused by severe MDR-AB bacteremia [45]. However, the risk of inappropriate early antibacterial treatment is significantly increased for bloodstream infections caused by MDR-AB [46]. In our study, all patients received improper empirical treatment. Due to the long time needed for taking blood culture, doctors usually use or change antibacterial drugs according to the symptoms and other auxiliary examinations. Although all strains are resistant to carbapenems, carbapenems are still important therapeutic options for treating MDR-AB infections in China.

In this study, the most sensitive antibacterial drugs were tigecycline and polymyxin B. However, the Chinese expert consensus on polymyxins in the clinical practice was not introduced until 2019 [12]. Clinicians usually lack medication experience and medication guidelines before this, which made the drug not widely used in mainland China. Several studies have shown that polymyxin B has a certain effect on infections caused by MDR-AB [47, 48]. Still, due to the small number of our cases, further studies are needed.

The usage rate of tigecycline is much higher than that of polymyxin B. Although the use of tigecycline does not improve the prognosis of patients, the combination of cefoperazone/sulbactam and tigecycline is still the most effective option. It has been reported that the combination of cefoperazone/sulbactam and tigecycline has synergistic effect in vitro [49, 50]. This combination can effectively reduce the inflammatory response of patients with MDR-AB pulmonary infection and improve patients' prognosis32169149. Our data suggest that this combination may also be suitable for the treatment of MDR-AB-induced bloodstream infections.

The pathogenicity of ST2 isolates was evaluated. ST2 isolates from the survival and non-survival group isolates showed no statistical difference in the biofilm formation, serum resistance, and virulence models. *A. baumannii* is a hypovirulent opportunistic pathogen [51]. Nevertheless, in this study, we found that the virulence and serum resistance of ST2 was significantly higher than that of the model strain. Because ST2 has multidrug resistance and high pathogenicity, it is difficult to evaluate the respective contributions of virulence and drug resistance in the infection process.

This study has several limitations. Firstly, it was conducted in a single center. Secondly, although cefoperazone/sulbactam and tigecycline were associated with lower mortality, the sample size was small. These findings need to be further validated by a larger multicenter study with a larger sample size.

## Conclusion

ST2 is the dominant ST type of MDR-AB causing bloodstream infection. BSI caused by ST2 *A. baumannii* strains represents a challenge for physicians, considering the high mortality associated with the infections. A priori prediction of resistance is difficult as the cultivation of *A. baumannii* from bloodstream infection takes a long time. Empiric coverage for ST2 strains is likely required even before determining antimicrobial susceptibility in critically ill patients. The combination of cefoperazone/sulbactam and tigecycline may be used as an effective treatment option.

## Data Availability

All data generated or analyzed during this study are included in this manuscript.

## Abbreviations

BSI: Bloodstream infection
MDR-AB: Multidrug-resistant *A. baumannii*
ST: Sequence type
WHO: World Health Organization
MLST: Multi-locus sequence typing
OR: Odds ratio
CIs: Confidence intervals
MICs: Minimum inhibitory concentrations
CFUs: Colony-forming units
SP-SDS: Single plate-serial dilution spotting
CLSI: Clinical and Laboratory Standards Institute
PBS: Phosphate-buffered saline
NHS: Normal human serum
